# *Microliciadeflexa* and *M.johnwurdackiana* (Melastomataceae), two new species from the Brazilian Cerrado

**DOI:** 10.3897/phytokeys.181.70949

**Published:** 2021-09-13

**Authors:** Rosana Romero, Rodrigo Valentim

**Affiliations:** 1 Instituto de Biologia, Universidade Federal de Uberlândia, Rua Ceará, s.n., 38400-902, Uberlândia, Minas Gerais, Brazil Universidade Federal de Uberlândia Uberlândia Brazil; 2 Programa de Pós-Graduação em Biologia Vegetal, Instituto de Biologia, Universidade Federal de Uberlândia, Rua Ceará, s.n., 38400-902, Uberlândia, Minas Gerais, Brazil Universidade Federal de Uberlândia Uberlândia Brazil

**Keywords:** Endemism, Microlicieae, Minas Gerais, Goiás, taxonomy

## Abstract

*Microliciadeflexa***sp. nov.** and *M.johnwurdackiana***sp. nov.** are described, illustrated and an updated identification key for the species of *Microlicia* from Clube Caça e Pesca Itororó de Uberlândia is provided. *Microliciadeflexa* occurs in Minas Gerais and Goiás States and is characterised by its linear-lanceolate and deflexed sepal on flower and immature fruit, long pedicel and indumentum of glandular trichomes, mixed with spherical, golden glands. *Microliciajohnwurdackiana* is endemic to Uberlândia and characterised by having indumentum of setose trichomes and spherical, golden glands, magenta petal with greenish abaxial surface at the apex and dimorphic stamens with bicolorous and tetrasporangiate anthers.

## Abstract (pt)

*Microliciadeflexa***sp. nov.** e *M.johnwurdackiana***sp. nov.** são descritas, ilustradas e uma chave de identificação atualizada para as espécies de *Microlicia* do Clube Caça e Pesca Itororó de Uberlândia é fornecida. *Microliciadeflexa* ocorre nos estados de Minas Gerais e Goiás, e é caracterizada pela sépala linear-lanceolada e deflexa na flor e no fruto imaturo, pedicelo longo e indumento com tricomas glandulares misturados com glândulas esféricas, douradas. *Microliciajohnwurdackiana* é endêmica de Uberlândia, e caracterizada pelo indumento de tricomas setosos e glândulas esféricas, douradas, pétala magenta com a face abaxial esverdeada apenas no ápice e estames dimórficos com anteras bicolores e tetraesporangiadas.

## Introduction

*Microlicia* D.Don is a predominantly Brazilian genus composed of ca. 240 species (Versiane et al. 2021), with most species occurring in the campo rupestre from Minas Gerais, Bahia and Goiás States (Romero 2003a, b; Romero et al. 2020) and about 49 species occurring in the cerrado phytophysiognomy. The species are generally characterised by solitary flowers or, in dichasia, reduced to one flower, with five to six occasionally nine petals, a superior or semi-inferior ovary with three to six locules and capsules dehiscing longitudinally from the apex to the base (basipetal) or from the base to the apex (acropetal) (see Versiane et al. 2021).

The Cerrado domain comprises approximately 23% of the central portion of Brazil and is represented by savannah and forest vegetation (Ribeiro and Walter 2008). Despite its rich and diverse flora (Klink and Machado 2005), the Cerrado has been threatened by economic development for several decades and has been classified as a biodiversity hotspot for more than 20 years (Myers et al. 2000).

The Triângulo Mineiro in western Minas Gerais with a heterogeneous landscape due to its different vegetation types, is included within the Cerrado domain (Novais 2011). Its central portion has an important fragment of vegetation inside a private club, the Clube Caça e Pesca Itororó de Uberlândia, in Uberlândia Municipality. This area has 127 hectares of well-preserved vegetation composed of cerrado, campo sujo, vereda, forest (Apolinário and Schiavini 2002) and a considerable area of campo úmido and campo limpo (Bacci et al. 2016). The inventory of the Melastomataceae from Clube Caça e Pesca Itororó de Uberlândia catalogued 28 species (Bacci et al. 2016); however, the authors were unable to fit some collections of *Microlicia* under any of the existing names in the genus. In this present study, we analysed these collections and confirm that two were undescribed species. Furthermore, from some of the collections from Serra dos Pireneus, Goiás State and Catas Altas, Minas Gerais, one was also recognised as a new species, increasing its geographic distribution. The two new species are described and morphologically compared with similar species. Information about their geographic distribution and conservation status and images of the morphological structures are provided. In addition, the identification key for the species of *Microlicia* in the Clube Caça e Pesca Itororó de Uberlândia is updated.

## Methods

This study was based on analysis of *Microlicia* specimens deposited mainly at HUFU, with duplicates at BHCB, HRCB, NY, OUPR, RB, UFG and US Herbaria (acronyms according to Thiers 2021) and on field observations in the Clube Caça e Pesca Itororó de Uberlândia. The general morphological terminology followed Radford et al. (1986) and the indumentum followed Wurdack (1986) and Silva et al. (2018). Distribution data were plotted on a map using the application ArcMap ver. 10.5.1. in ArcGis Desktop (ESRI 2017). The area of occupancy (AOO) and extent of occurrence (EOO) were calculated using GeoCAT georeferenced data from cited collections (Bachman et al. 2011). Recommended conservation assessments were based on the IUCN Red List Categories and Criteria (IUCN 2019). Images of vegetative and reproductive structures were obtained using a digital camera coupled to a Zeiss stereoscopic microscope and organised on Adobe Photoshop CS6.

## Taxonomic treatment

### Microlicia deflexa

Taxon classificationPlantaeMyrtalesMelastomataceae

R.Romero & Valentim sp. nov.

A36C6E4C-E88C-55BE-8D06-2C4F22ADC79F

urn:lsid:ipni.org:names:77219658-1

Figs 1 , 2

#### Type.

Brazil. Minas Gerais: Uberlândia, Clube Caça e Pesca Itororó de Uberlândia, 9 June 2011 (fl, fr), *A.F.A. Versiane et al. 20* (holotype: HUFU!; isotypes: BHCB!, HRCB!, OUPR!, UFG!).

**Figure 1.**
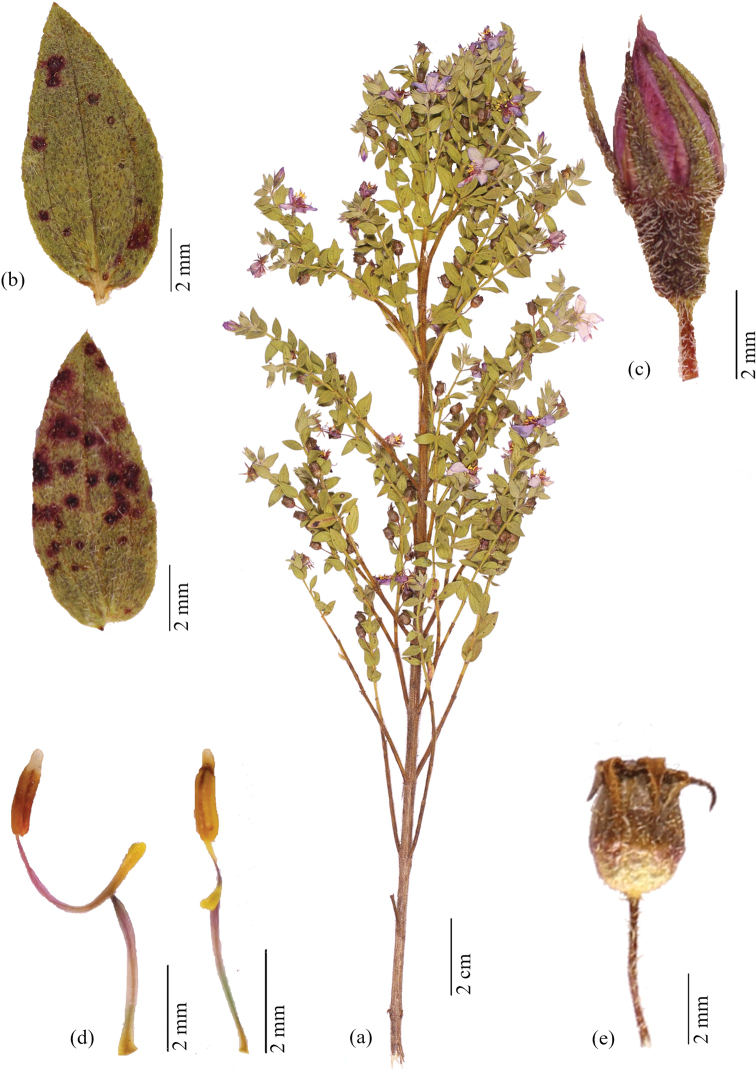
*Microliciadeflexa* R.Romero & Valentim **A** flowering branch **B** leaf abaxial surface (up) and adaxial surface (down) **C** floral bud **D** larger (antesepalous) stamen (on the left) and smaller (antepetalous) stamen (on the right) **E** immature fruit enveloped by the hypanthium with deflexed sepals (**A, E***R. Romero 8690***B–D***A.F.A. Versiane 20*).

#### Diagnosis.

*Microliciadeflexa* is recognised by the deflexed and linear-lanceolate sepals on flowers and immature fruits, long pedicels (3–5 mm long) and indumentum of glandular trichomes, glands sometimes caducous, mixed with spherical, golden glands covering branch, leaf, pedicel, hypanthium and sepal.

**Figure 2.**
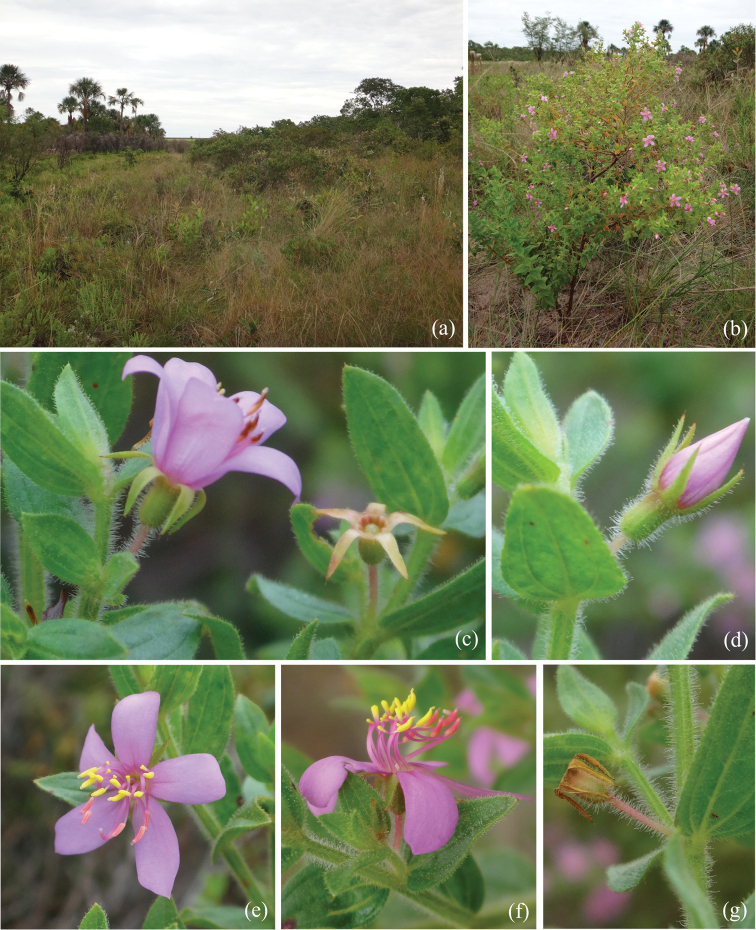
*Microliciadeflexa* R.Romero & Valentim **A** cerrado landscape at Uberlândia, Triângulo Mineiro, Brazil, the type locality of *M.deflexa***B** habit **C** details of the deflexed sepals on the flower and immature fruits **D** flower bud **E** flower in frontal view **F** flower in lateral view **G** immature fruit. Photos: Rosana Romero.

#### Description.

Subshrub or shrub, 0.3–0.8 m tall, erect, multi-branched. Stem terete, glabrous, brownish. Branch fastigiate, younger branch quadrangular, green, densely covered with glandular trichomes 0.4–0.8 mm long and spherical, golden glands, older branch terete, brownish, leafless with age. Leaf ascending, not imbricate; petiole up to 0.6 mm long, leaf rarely sessile; blade 3–18.5 × 1.5–10.5 mm, leaf larger in the main branch, concolorous (when dry), green, sometimes with magenta or yellow-brownish spots on both surfaces, chartaceous, oblong to ovate-oblong, sometimes elliptic, rarely lanceolate, acute at the apex, with a terminal setose trichome 0.1–0.4 mm long, base rounded, margin slightly sinuous to serrate-dentate, glandular-ciliate, both surfaces with a dense indumentum of glandular trichomes 0.4–0.8 mm long, glands sometimes caducous, mixed with spherical, golden or sometimes vinaceous glands, 3–5-veined, veins conspicuously visible on both surfaces, impressed on the adaxial surface, thickened and prominent on abaxial surface. Flower 5-merous, solitary, terminal or lateral, perianth actinomorphic; pedicel 3–5 mm long; hypanthium 1.8–2.3 × 1–2 mm, green, cylindrical, with a dense indumentum of glandular trichomes 0.1–0.8 mm long, glands sometimes caducous, mixed with spherical, golden glands, calyx tube ca. 0.2 mm long, sepal 3–3.2 × 0.5–1.2 mm, longer than the length of the hypanthium, deflexed, green, sometimes magenta at the apex (when dry), linear-lanceolate, acute at the apex, with a terminal setose trichome 0.1–0.4 mm long, with glandular trichomes 0.1–0.3 mm long, glands sometimes caducous, mixed with spherical, golden glands; petal 6–7 × 3–4.5 mm, pinkish, oblong or obovate, acute at the apex, margin entire, glabrous; stamen 10, dimorphic, anther bicolorous, tetrasporangiate; larger (antesepalous) stamen with filament 2.5–3.7 mm long, lilac, pedoconnective 1.5–2.5 mm long, lilac, ventral appendage 0.7–1.2 mm long, yellow, retuse or sometimes slightly bilobed at the apex, anther 1.8–2.2 mm long including beak, pinkish, sometimes with yellow spots, oblong, beak 0.2–0.4 mm long, white; smaller (antepetalous) stamen with filament 2.4–3.7 mm long, lilac, pedoconnective 0.7–1.2 mm long, lilac, ventral appendage 0.2–0.4 mm long, yellow, retuse at the apex, anther ca. 1.5 mm long including beak, yellow, oblong, beak 0.2–0.4 mm long, yellow; ovary 3–5-locular, sub-globose to terete, superior, glabrous; style 3.5–6.5 mm long, terete, slightly curved, pinkish, glabrous; stigma punctiform. Capsule 2.7–4.2 × 1.3–2.7 mm, brownish, globose, dehiscing into 3–5 valves from the apex, hypanthium enveloping the entire capsule and peeling off top to bottom as the fruit mature, columella deciduous; seed 0.2–0.4 × 0.1–0.3 mm, pale brown, oblong, testa foveolate.

#### Distribution and habitat.

**Figure 3.**
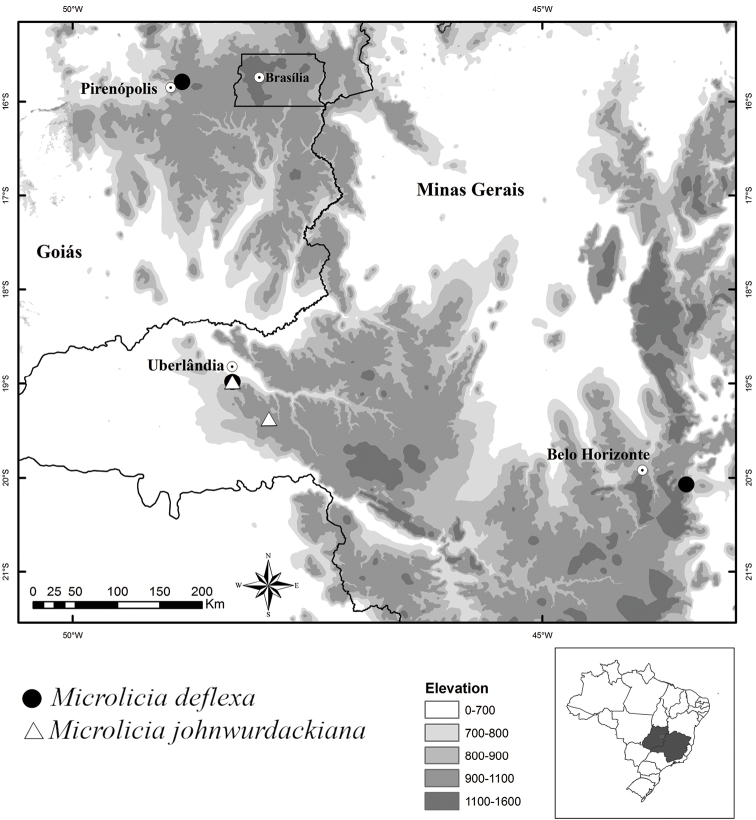
Geographical distribution of *Microliciadeflexa* and *Microliciajohnwurdackiana* in Minas Gerais and Goiás States, Brazil.

*Microliciadeflexa* is found in Minas Gerais, in Uberlândia, occurring in campo sujo near vereda, at ca. 860 m elevation and in Catas Altas, in a shaded area of cerrado, at 1085 m elevation. It also occurs in Goiás, in Cocalzinho de Goiás, in campo sujo near vereda, at 1220 m elevation (Fig. 3).

#### Conservation status.

*Microliciadeflexa* has a restricted area of occupancy (AOO = 12 km^2^) and should be preliminarily assessed as Endangered [(EN) B2ab (iii)], following the IUCN (2019) guidelines. Only the populations in Cata Altas are protected since it occurs inside the Reserva Patrimônio Natural Santuário do Caraça.

#### Phenology.

Flowers have been collected in April and from July to September and fruits from July to October.

#### Etymology.

The specific epithet “deflexa” refers to the sepal characteristically deflexed on flower and immature fruit i.e. turned abruptly downwards.

#### Discussion.

*Microliciadeflexa* is morphologically similar to *M.serpyllifolia* D.Don, which occurs in Rio de Janeiro, Minas Gerais, Goiás, Bahia and Distrito Federal (Silva and Romero 2008, as *M.fulva*; Romero and Woodgyer 2015; Romero et al. 2020) in campo rupestre, campo limpo and cerrado. Both species have long pedicel and sepal, dimorphic stamen with bicolorous and tetrasporangiate anther. *Microliciaserpyllifolia* differs in having a very peculiar velutinous indumentum composed of thin, short and soft trichomes covering branch, leaf, pedicel, hypanthium and sepal mixed with spherical glands (vs. glandular indumentum mixed with spherical, golden glands in *M.deflexa*). The sepal in *M.serpyllifolia* is triangular and ascending (vs. linear-lanceolate and deflexed). *Microliciadeflexa* also bears some resemblance to *Microliciahelvola* (Spreng.) Triana, which occurs in Goiás, Mato Grosso do Sul, Mato Grosso and Minas Gerais States (Romero et al. 2020; Versiane et al. 2020) in campo limpo, campo sujo and the edges of vereda and swamp areas (Bacci et al. 2016; Versiane et al. 2016) and less frequently in cerrado rupestre (Machado and Romero 2020). *Microliciadeflexa* and *M.helvola* are subshrub or shrub with sessile leaf or with a short petiole, acute at the apex, rounded at the base and 3–5-veined. Furthermore, both species have pinkish petal and dimorphic stamens with bicolorous anthers. However, *M.helvola* differs in having an indumentum of setose trichomes mixed with spherical glands (vs. glandular trichomes mixed with spherical glands in *M.deflexa*), urceolate and 10-striate hypanthium (vs. cylindrical and not striate), sepal shorter than the hypanthium length (vs. sepal longer than the hypanthium length) and polysporangiate anther (vs. tetraesporangiate). *Microliciadeflexa* also resembles *M.phlogiformis* (DC.) Versiane & R.Romero, which also occurs in the Clube Caça e Pesca Itororó de Uberlândia, in campo úmido or inside the vereda on waterlogged soil (Bacci et al. 2016). The indumentum, the sepals and the long pedicel of both species are similar; however, *M.phlogiformis* differs in having flowers in dichasia (vs. solitary) and ascending sepals (vs. deflexed). Table 1 includes additional features comparing *M.helvola*, *M.phlogiformis* and *M.serpyllifolia* to *M.deflexa*.

**Table 1.** Comparative features between *Microliciadeflexa* and relatives.

Characters/species	* M. deflexa *	* M. helvola *	* M. phlogiformis *	* M. serpyllifolia *
Petiole (mm)	Absent or up to 0.6	Absent or up to 0.5	Absent or 2–5	Absent or ca. 0.4
Pedicel (mm)	3–5	0.7–1	2–9	1.3–3
Leaf trichomes	Glandular trichomes, and spherical glands	Setose and spherical glands	Glandular trichomes	Setose and spherical glands
Hypanthium shape	Cylindrical	Urceolate	Urceolate	Campanulate
Sepal length (mm)	3–3.2	1–2	3.5–4	1.3–4
Sepal	Deflexed	Ascending	Ascending	Ascending
Anther sporangia	Tetrasporangiate	Polysporangiate	Tetrasporangiate	Tetrasporangiate
Distribution	Minas Gerais, Goiás	Goiás, Mato Grosso do Sul, Mato Grosso, Minas Gerais	Paraná, Espírito Santo, Minas Gerais, Rio de Janeiro, São Paulo, Goiás, Mato Grosso do Sul, Mato Grosso, Bahia, Distrito Federal	Rio de Janeiro, Minas Gerais, Goiás, Bahia, Distrito Federal

#### Additional specimens examined (paratypes).

Brazil. Goiás: Cocalzinho de Goiás, estrada para plantação de eucalipto final da estrada, 26 April 2012 (fl), *J.N. Nakajima et al. 5083* (BHCB!, HRCB!, HUFU!, NY!, RB!, UFG!, US!). Minas Gerais: Catas Altas, Reserva Patrimônio Natural Santuário do Caraça, trilha para a cascatinha, 18 October 2016 (fr), *M. Castro et al. 180* (BHCB!, HUFU!); Uberlândia, Clube Caça e Pesca Itororó de Uberlândia, 21 March 2011, *A.F.A. Versiane et al. 2* (HUFU!, SP!, UEC!); idem, 6 April 2011 (fl), *A.F.A. Versiane 10* (HUFU!, NY!, OUPR!, RB!); idem, 12 July 2011 (fl, fr), *A.F.A. Versiane et al. 29* (HUFU!, HRCB!); idem, 12 July 2011 (fl, fr), *A.F.A. Versiane et al. 30* (HUFU!, K!, P!); idem, 12 July 2011 (fl, fr), *A.F.A. Versiane et al. 32* (HUFU!); idem, 26 August 2015 (fl, fr), *R. Romero et al. 8690* (HUFU!, OUPR!); idem, 9 September 2015 (fl, fr), *R. Romero et al. 8693* and *8695* (HUFU!); idem, 4 February 2017 (fl), *A.P.S. Caetano 52* (HUFU!); idem, 10 May 2018 (fl, fr), *R.V. Brito et al. 37* (HUFU!).

### Microlicia johnwurdackiana

Taxon classificationPlantaeMyrtalesMelastomataceae

R.Romero & Valentim sp. nov.

0C14CE31-3E95-533E-80AB-D3DA4E601E80

urn:lsid:ipni.org:names:77219659-1

Figs 4 , 5

#### Type.

Brazil. Minas Gerais: Uberlândia, 26 August 2015 (fl, fr), *R. Romero et al. 8687* (holotype: HUFU!; isotypes: BHCB!, K!, OUPR!, P!, RB!, UEC!).

**Figure 4.**
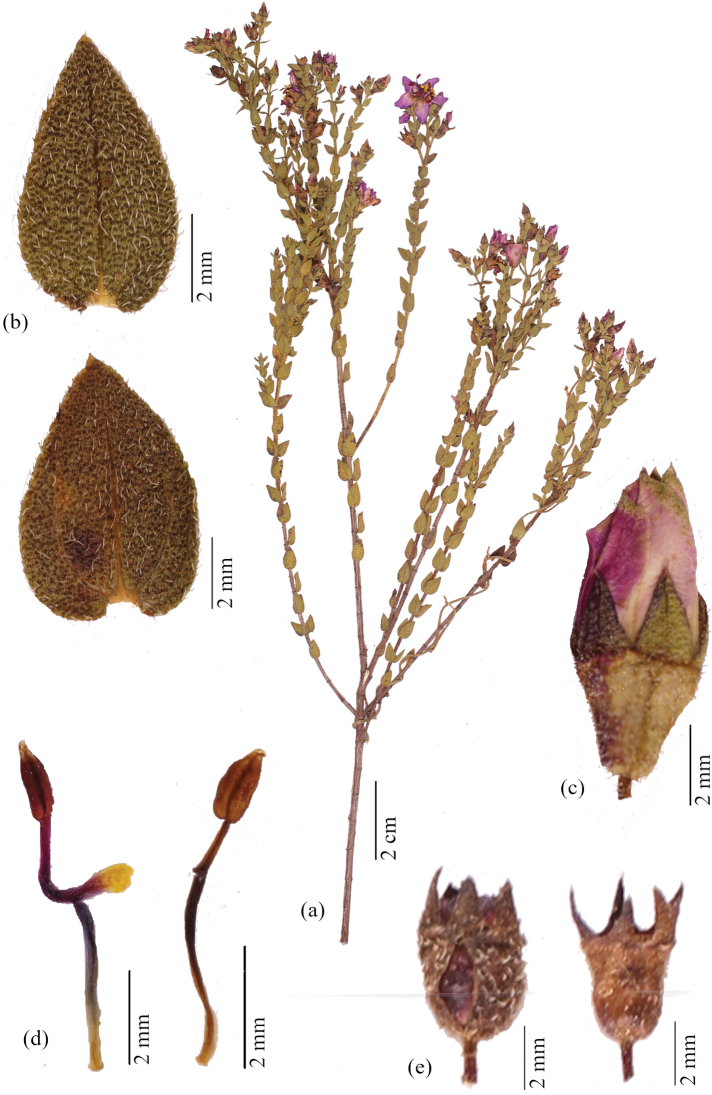
*Microliciajohnwurdackiana* R.Romero & Valentim **A** flowering branch **B** leaf abaxial surface (up) and adaxial surface (down) **C** floral bud **D** larger (antesepalous) stamen (on the left) and smaller (antepetalous) stamen (on the right) **E** capsule in two stages: immature enveloped by the hypanthium (on the right) and mature with hypanthium peeling off (on the left) (**A, E***A.F.A. Versiane et al. 57***B–D***M.L. Viana 4*).

#### Diagnosis.

*Microliciajohnwurdackiana* is recognised by its dense indumentum of setose trichomes and spherical, golden glands covering branch, leaf, pedicel, hypanthium and sepal, magenta petal with greenish apex on the abaxial surface and bicolorous and tetraesporangiate anther.

**Figure 5.**
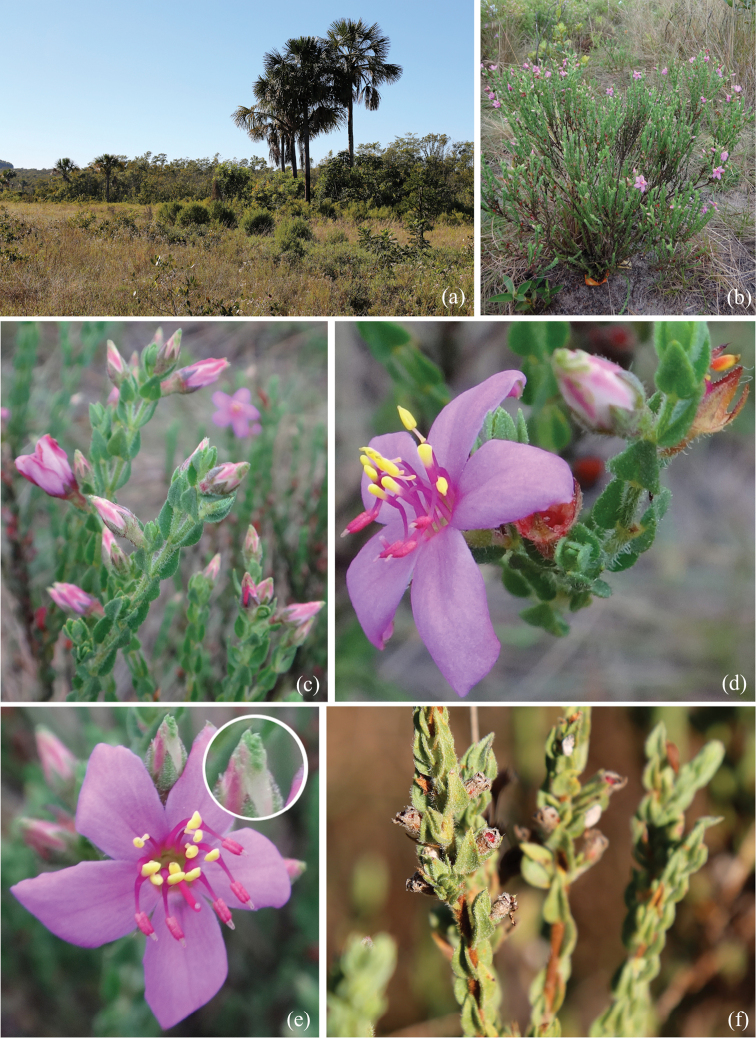
*Microliciajohnwurdackiana* R.Romero & Valentim **A** cerrado landscape at Uberlândia, Triângulo Mineiro, Brazil, the type locality of *M.johnwurdackiana***B** habit **C** flowering branches **D** flower in lateral view **E** flower in front view, detail of a greenish apex in the petals in the flower bud **F** immature fruits. Photos: Rosana Romero.

#### Description.

Subshrub or shrub, 0.3–1 m tall, erect, multi-branched. Stem terete, brownish. Branch fastigiate, younger branch quadrangular, green, densely covered of setose, pale trichomes 0.2–0.3 mm long mixed with spherical, golden glands, older branch sub-quadrangular, brownish, leafless with age. Leaf ascending, not imbricate; petiole ca. 0.3 mm long, leaf rarely sessile; blade 3.5–9.5 × 1.7–7 mm, leaf with the same size in the main and lateral branches, concolorous (when dry), green-brownish, chartaceous, ovate, acute at the apex, base rounded, margin crenulate, setose-ciliate, both surfaces with a dense indumentum of setose, pale trichomes 0.2–0.3 mm long mixed with spherical, golden glands, 3-veined, rarely 5-veined. Flower 5-merous, solitary, terminal or lateral, perianth actinomorphic; pedicel 0.8–1.2 mm long; hypanthium 2.3–3 × ca. 1.5 mm, light green, cylindrical, with a dense indumentum of setose, pale trichomes 0.2–0.3 mm long mixed with spherical, golden glands; calyx tube ca. 0.3 mm long; sepal 1.5–2 × 1–1.5 mm, vinaceous, triangular, with a dense indumentum of setose, pale trichomes 0.2–0.3 mm long mixed with spherical, golden glands, acute at the apex, with a terminal setose trichome ca. 0.2 mm long; petal 4.5–8 × 3.5–5 mm, magenta, greenish at the apex on the abaxial surface, obovate, acute or asymmetrically acuminate at the apex, margin entire, with sparse, spherical, golden glands at the apex; stamen 10, dimorphic, anther bicolorous, tetraesporangiate; larger (antesepalous) stamen, with filament 2.5–3 mm long, vinaceous, pedoconnective 1.8–2.5 mm long, vinaceous, ventral appendage ca. 1 mm long, with proximal half magenta, distal half yellow, obtuse at the apex, anther 2–2.5 mm long including beak, vinaceous, ovate-oblong, beak 0.4–0.6 mm long, white; smaller (antepetalous) stamen with filament 2–3 mm long, vinaceous, pedoconnective ca. 0.8 mm long, yellow, ventral appendage ca. 0.1 mm long, yellow, retuse at the apex, anther 1.5–1.8 mm long including beak, yellow, ovate-oblong, beak 0.3–0.5 mm long, yellow; ovary 3-locular, ovate to ovate-elliptic, superior, glabrous; style 4–4.5 mm long, magenta, terete, slightly curved, glabrous; stigma punctiform. Capsule 4–4.5 × 2–2.5 mm brownish to reddish, globose, dehiscing into 3-valves from the apex, hypanthium and sepals enveloping the entire capsule and peeling off top to bottom as the fruit mature, columella deciduous; seed 0.3–0.5 × 0.2–0.3 mm, brownish or reddish, oblong, testa foveolate.

#### Distribution and habitat.

*Microliciajohnwurdackiana* is endemic to Uberlândia, city of the Triângulo Mineiro, western Minas Gerais, Brazil. It occurs at Clube Caça e Pesca Itororó de Uberlândia in campo sujo near to vereda, on sandy soil, and in a private area in campo úmido with murundus (see Paulino et al. 2015) in the upper course of the Bacia do Rio Uberabinha, about 850 m elevation (Fig. 3).

#### Conservation status.

*Microliciajohnwurdackiana* has a restricted area of occupancy (AOO = 12 km^2^) and, according to the IUCN Categories and Criteria (IUCN 2019), we recommend a conservation status of Critically Endangered [CR B1ab (iii) + 2ab (iii)]. According to the Brazilian Forest Code (Law 12.651/2012), the vereda in rural or urban areas are permanent preservation areas (APP). However, both localities, where *M.johnwurdackiana* occurs, are not protected by any conservation unit. Large populations occur at Clube Caça e Pesca Itororó de Uberlândia, a well-preserved vegetation fragment, located in the urban area of Uberlândia, recognised in 1992 as Reserva Particular de Patrimônio Natural (RPPN) (IBAMA 1992). However, the ordinance was revoked by the same Institute (IBAMA 2000) for lack of proper documentation, making this area vulnerable, except for the vereda and its surroundings, which is a permanent preservation area. Nevertheless, in recent years, this area has been heavily impacted by periodic fires, predatory collections, real estate speculation and the opening of trails for cyclists in the interior of the cerrado. The other area of occurrence is private property that has been seriously affected by the expansion of agriculture, invasion of exotic *Pinus* species, removal of refractory clay and frequent burning caused by farmers. As a result of so many threats, the civil society from Uberlândia has made an effort, through the non-governmental organisation Angá (Associação para a Gestão Socioambiental do Triângulo Mineiro), for part of this area to become a permanent preservation area (P.K.B. Hemsing, pers. comm.).

#### Phenology.

Flowers and fruits have been collected from March to May and from July to December.

#### Etymology.

The specific epithet honours John Julius Wurdack (1921–1998), an American botanist who dedicated part of his life to studying the Melastomataceae family and described more than 900 species (see IPNI 2021). About 20 years ago, Wurdack examined the first collections of *Microlicia* made at Clube Caça e Pesca Itororó de Uberlândia (*Romero et al. 535* at US) and indicated that it was likely a new species.

#### Discussion.

*Microliciajohnwurdackiana* is similar to *M.hirticalyx* Romero & Woodgyer, which is endemic to the south of the Espinhaço Range, Minas Gerais State, occurring in campo rupestre (Romero and Woodgyer 2011). Both species have a dense indumentum of setose trichomes mixed with spherical, golden glands covering branch, leaf, hypanthium and sepal, 5-merous flower, solitary, terminal and lateral, dimorphic stamens with bicolorous and tetrasporangiate anthers. *Microliciahirticalyx* differs in having oblong-campanulate hypanthium with patent trichomes 0.5–1.5 mm long (vs. cylindrical, ascending trichomes 0.2–0.3 mm long in *M.johnwurdackiana*), sepal 2–3.5 mm long (vs. 1.5–2 mm long) and petal apiculate at the apex (vs. acute or asymmetrically acuminate, not apiculate). *Microliciajohnwurdackiana* also bears some resemblance to *M.fasciculata* Martius ex Naudin and *M.polystemma* Naudin. *Microliciafasciculata* occurs in São Paulo, Minas Gerais, Goiás, Bahia and Distrito Federal (Silva and Romero 2008; Romero et al. 2020) in campo rupestre, cerrado, campo limpo, campo sujo and campo úmido, while *M.polystemma* occurs in São Paulo, Minas Gerais, Goiás and Distrito Federal (Silva and Romero 2008; Romero and Woodgyer 2015; Bacci et al. 2016; Romero et al. 2020) in campo rupestre and campo úmido. Both species are similar to *M.johnwurdackiana* in having setose trichomes and spherical glands covering the branch, leaf, pedicel, hypanthium and sepal. However, *M.fasciculata* has a villous indumentum with white trichomes that give a glaucous tonality to the plant (vs. setose indumentum with pale trichomes in *M.johnwurdackiana*), campanulate hypanthium (vs. cylindrical), petal entirely pink (vs. magenta, greenish at the apex on the abaxial surface), rounded at the apex (vs. acute or asymmetrically acuminate), ciliate-glandular margin (vs. with sparse, spherical, golden glands only at the apex) and polysporangiate anther (vs. tetrasporangiate). *Microliciapolystemma* differs in having campanulate hypanthium (vs. cylindrical in *M.johnwurdackiana*), yellow stamens, sometimes with pink spots in the anther (vs. vinaceous anther in the antesepalous whorl and yellow in the antepetalous one) and ovate-triangular sepal with a conspicuous setose trichome ca. 0.8 mm long at the apex (vs. triangular, trichome ca. 0.2 mm long). Table 2 includes additional features comparing *M.hirticalyx*, *M.fasciculata* and *M.polystemma* to *M.johnwurdackiana*.

**Table 2.** Comparative features between *Microliciajohnwurdackiana* and relatives.

Characters/species	* M. fasciculata *	* M. hirticalyx *	* M. johnwurdackiana *	* M. polystemma *
Hypanthium shape	Campanulate	Oblong-campanulate	Cylindrical	Campanulate
Sepal length (mm)	1.6–2.3	2–4	1.5–2	2.5–4.4
Sepal shape	Triangular	Narrowly triangular	Triangular	Ovate-triangular
Petal apex	Acute, asymmetrically acute, or rounded	Apiculate	Acute or asymmetrically acuminate	Rounded
Anther, numbers of sporangia	Polysporangiate	Tetrasporangiate	Tetrasporangiate	Tetrasporangiate
Anthers colours	Bicolorous, rarely concolorous	Bicolorous	Bicolorous	Concolorous
Distribution	São Paulo, Minas Gerais, Goiás, Bahia, Distrito Federal	Minas Gerais	Minas Gerais	São Paulo, Minas Gerais, Goiás, Distrito Federal

#### Additional specimens examined (paratypes).

Brazil. Minas Gerais: Uberlândia, Clube Caça e Pesca Itororó de Uberlândia, 22 October 1993 (fl, fr), *R. Romero et al. 535* (HUFU!, US!); idem, 30 November 1993 (fl, fr), *R. Romero & A.A. Arantes 553* (HUFU!); idem, 1 December 1993 (fl, fr), *R .Romero & J.N. Nakajima 559* (HUFU!); idem, 22 March 1994 (fl, fr), *R. Romero 780* (HUFU!, K!); idem, 17 May 1994 (fl), *R. Romero et al. 1004* (HUFU!); idem, 24 May 1994 (fr), *R. Romero & A.A. Arantes 1013* (BHCB!, HUFU!); idem, 9 October 1998 (fl, fr), *G.M. Araújo s.n*. (HUFU 17845!); idem, 4 December 1998 (fl), *A.F. Amaral et al 1419* (HUFU!); idem, 5 March 1999, *A.A.A. Barbosa 1912* (HUFU!, SP!); idem, 29 November 2002 (fl), *A.A.A. Barbosa s.n*. (HUFU 31783!); idem, 14 April 2009 (fl, fr), *R. Romero et al. 8212* (HUFU!, UEC!); idem, 26 May 2011 (fl, fr), *A.F.A. Versiane & L.F. Bacci 17* (HUFU!, P!, US!); idem, 27 July 2011 (fl, fr), *A.F.A. Versiane & L.F. Bacci 34* (HUFU!, RB!); idem, 1 September 2011 (fl, fr), *A.F.A. Versiane et al. 39* (HUFU!, K!, P!, SP!, US!); idem, 1 November 2011 (fl, fr), *A.F.A. Versiane et al. 57* (HUFU!, SP!, UEC!, UFG!); idem, 22 August 2012 (fl, fr), *A.F.A. Versiane et al. 243* (HUFU!); idem, 26 October 2015, *R. Romero 8687* (HUFU!); idem, 6 November 2015 (fl, fr), *F.L. Contro et al. 169* (HUFU!); idem, 6 November 2015 (fl, fr), *J.N. Nakajima 5100* (HUFU!); idem, 9 May 2016 (fl, fr), *R. Romero 8825* (HUFU!, RB!); idem, 16 March 2017 (fl, fr), *P.K.B. Hemsing et al. 564* (HUFU!) and *571* (HUFU!); idem, 30 October 2017 (fl, fr), *J. Santiago et al. s.n.* (HUFU 75665!); idem, 30 October 2017 (fl, fr), *R.G. Clemente et al. s.n*. (HUFU 75682!); idem, 9 March 2018 (fr), *M.L. Viana 4* (HUFU!); idem, 7 May 2018 (fl, fr), *F. L. Jesus et al. s.n*. (HUFU 76820!); idem, 7 May 2018 (fl, fr), *T. R. Leal & V. R. Teixeira s.n.* (HUFU 76818!); idem, 10 May 2018 (fl, fr), *R.V. Brito et al. 42* (HUFU!); Bacia do Rio Uberabinha, 19°22'33"S, 47°54'44"W, 9 October 2014 (fl, fr), *P.K.B. Hemsing & J.C. Aguilar 328* (BHCB!, HUFU!, OUPR!).

### Key to the species of *Microlicia* from Clube Caça e Pesca Itororó de Uberlândia, Minas Gerais State, Brazil

**Table:** 

1	Flower 6-merous; capsule dehiscing from the base	*** Microlicia macrantha ***
–	Flower 5-merous; capsule dehiscing from the apex	**2**
2	Anther polysporangiate	**3**
–	Anther tetrasporangiate	**4**
3	Leaf blade with glaucous, villous indumentum; hypanthium campanulate	*** Microlicia fasciculata ***
–	Leaf blade with greenish to yellowish, setose indumentum; hypanthium urceolate	*** Microlicia helvola ***
4	Petal white to pinkish-white or cream to white with pink vein	**5**
–	Petal pinkish or magenta	**6**
5	Leaf discolorous (darker adaxial surface), revolute at the margin; petiole 6–10 mm long; flower with a short pedicel (1–2 mm long)	*** Microlicia parviflora ***
–	Leaf concolorous (green), flat at the margin; petiole 2–3 mm long; flower with a long pedicel (6–9 mm long)	*** Microlicia phlogiformis ***
6	Main branch with leaf larger than in the lateral branches	**7**
–	Main and lateral branches with the same size leaf	**8**
7	Leaf blade cordate; hypanthium campanulate; sepal positioned horizontally on flower and immature fruit	*** Microlicia cordata ***
–	Leaf blade oblong to ovate-oblong, sometimes elliptic, rare lanceolate; hypanthium cylindrical; sepal deflexed on flower and immature fruit	*** Microlicia deflexa ***
8	Stamen with concolorous anther (yellow), sometimes with vinaceous spots; petal longer than 8.5 mm	*** Microlicia polystemma ***
–	Stamen with bicolorous anther (vinaceous and yellow); petal less than 8 mm	**9**
9	Leafblade with dense indumentum of setose trichomes and spherical glands; hypanthium cylindrical; petal with greenish abaxial surface at the apex	*** Microlicia johnwurdackiana ***
–	Leafblade with sparse indumentum of spherical glands; hypanthium campanulate; petal entirely magenta	*** Microlicia trembleyiformis ***

Bacci et al. (2016) catalogued seven species of *Microlicia* to Clube Caça e Pesca Itororó de Uberlândia: *M.cordata* (Spreng.) Cham., *M.fasciculata*, *M.helvola*, *M.polystemma*, *M.macrantha* Versiane & R.Romero (as *Lavoisieragrandiflora* A.St.-Hil. ex Naudin), *M.parviflora* (D.Don) Versiane & R.Romero (as *Trembleyaparviflora* D.Don) and *M.phlogiformis* (DC.) Versiane & R.Romero (as *Trembleyaphlogiformis* DC.) and we add here three more species: *M.deflexa*, *M.johnwurdackiana* and *M.trembleyiformis* Naudin.

## Supplementary Material

XML Treatment for Microlicia deflexa

XML Treatment for Microlicia johnwurdackiana
